# Aqueous humor metabolomic profiling identifies a distinct signature in pseudoexfoliation syndrome

**DOI:** 10.3389/fmolb.2024.1487115

**Published:** 2025-01-23

**Authors:** Arturs Zemitis, Juris Vanags, Theresa Schiemer, Kristaps Klavins, Guna Laganovska

**Affiliations:** ^1^ Department of Ophthalmology, Riga Stradins University, Riga, Latvia; ^2^ Pauls Stradins Clinical University Hospital, Clinic of Ophthalmology, Riga, Latvia; ^3^ Institute of Biomaterials and Bioengineering, Faculty of Natural Sciences and Technology, Riga Technical University, Riga, Latvia; ^4^ Baltic Biomaterials Centre of Excellence, Headquarters at Riga Technical University, Riga, Latvia

**Keywords:** pseudoexfoliation syndrome, cysteine, antiporter system X_c_^−^, arginine, tryptophan, oxidative stress, iron

## Abstract

**Purpose:**

PEXS was first described in 1917, yet its etiology still needs clarification. An imbalance between oxidants and antioxidants plays a significant role. PEXS leads to various ocular complications, including increased risk during cataract surgery due to weak zonules, lens dislocation, and reduced visual outcomes. Our study investigates whether metabolomics can provide insights into this ocular pathology.

**Methods:**

The study included 183 patients undergoing cataract surgery at Pauls Stradins Clinical University Hospital. 104 patients did not have PEXS, while 79 were diagnosed with the condition. Intraocular fluid samples from these patients were analyzed using targeted metabolite analysis, performed through HILIC liquid chromatography coupled with mass spectrometry detection.

**Results:**

The aqueous humor of PEXS patients contains statistically significant higher levels of cystine (*p* < 0.001), citrulline (*p* < 0.001), phenylalanine (*p* = 0.041), tyrosine (*p* = 0.025), serine (*p* = 0.030), arginine (*p* = 0.017), lactic acid (*p* = 0.055), tryptophan (*p* = 0.055), and creatinine (*p* = 0.022). These results suggest a potential link to ferroptosis.

**Conclusion:**

Ferroptosis is a form of programmed cell death characterized by iron-dependent LPO. The inhibition of the antiporter system X_c_^−^ leads to increased oxidative stress, suggesting that the changes seen in PEXS could be linked to ferroptosis. Our findings indicate that cysteine synthesis occurs via the transsulfation pathway, attributable to inhibiting the antiporter system X_c_^−^. Treatment of pseudoexfoliation should lower the oxidative stress inside the anterior chamber by reducing the uptake of PUFAs, lower iron levels, and cysteine supplementation.

## 1 Introduction

Finnish ophthalmologist John Gustaf Lindberg presented the first description of pseudoexfoliation syndrome (PEXS) in his doctoral thesis in 1917 (Grzybowski et al., 2019). However, the precise etiology of PEXS still needs to be determined. PEXS leads to the accumulation of extracellular material in various ocular tissues (Tuteja et al., 2024). It is believed that it is a systemic disorder that presents primarily with implications beyond just the eye. It is characterized by the gradual and chronic deposition and buildup of grayish-white material in various organs (Tuteja et al., 2023). Using transmission electron microscopy, similar fibers to PEX have been found in autopsy tissue specimens of the heart, lungs, liver, kidney, skin, and cerebral meninges (Schlötzer-Schrehardt et al., 1992). Clinically unilateral PEXS supports the notion that it is fundamentally a bilateral disorder, characterized by a markedly asymmetric clinical presentation (Hammer et al., 2001). An imbalance between oxidants and antioxidants may play a part in developing PEXS, a disease marked by faulty extracellular matrix (ECM) remodeling (Mastronikolis et al., 2022). PEXS impacts tissues made up of elastic fibers, like the walls of blood vessels, the trabecular meshwork, and the lamina cribrosa (Vazquez and Lee, 2014). Oxidative stress and inflammation are pivotal contributors to the pathogenesis of PEXS (Botling Taube et al., 2019). The predominant presence of elastic fiber epitopes, particularly elastic microfibrillar components such as elastin, vitronectin, amyloid P, fibrillin-1, MAGP-1, emilin, LTBP-1, and LTBP-2, supports the current theory that PEXS is a form of elastosis that primarily affects elastic microfibrils (Ritch and Schlötzer-Schrehardt, 2001). The ECM is a complex three-dimensional network comprising collagen, elastin, fibronectin, hyaluronic acid, proteoglycans, and glycoproteins. This intricate structure provides essential support to tissues by encapsulating cells and maintaining hydration and pH homeostasis (Roma-Rodrigues et al., 2019).

Cardiovascular conditions significantly associated with PEXS included ischemic heart disease, aortic aneurysms, and homocystinuria (Siordia J. et al., 2016). The association between PEX and ischemic heart disease was statistically significant, with a *p*-value of 0.045 (Siordia JA. et al., 2016). The prevalence of Alzheimer’s-related dementia is elevated in patients with PEXS (Cumurcu et al., 2013). There is a hypothesis positing PEXS as a systemic disorder (Amari et al., 1994). These changes are only marginally functional and not life-threatening (Slettedal et al., 2015). PEXS leads to a variety of ocular complications. It is associated with an augmented propensity for complications during cataract surgery (Shivkumar et al., 2022). It is linked to complicated initial cataract surgery due to weak zonules and late-in-the-bag dislocation (Vanags and Laganovska, 2020). These complications reduce visual outcomes after cataract operations (Vazquez-Ferreiro et al., 2021). The presence of material on the zonules might account for the clinically observed phenomenon of zonular weakness and the subsequent subluxation or dislocation of the lens (Yüksel and Yılmaz Tuğan, 2023). Proteomic studies of the lens zonule indicate that human zonules are predominantly composed of non-collagenous acidic glycoproteins, with fibrillin-1 being the most abundant component (Pan et al., 2023). Patients with PEXS who underwent cataract surgery by trainees had higher rates of posterior capsular rupture with vitreous loss and worse visual outcomes compared to those operated on by experienced consultants (Singh et al., 2021). PEXS is the leading risk factor for secondary open-angle glaucoma called pseudoexfoliative glaucoma (PEXG). Approximately 30%–50% of individuals with PEXS progress to developing glaucoma (Ritch, 1994). It is a significant cause of blindness on a global scale (Sahay et al., 2022). It has been studied that patients with PEXG benefit from a greater intraocular pressure reduction after cataract surgery compared to controls (Ramezani et al., 2021).

Metabolomics is a pivotal aspect of our biological existence, serving as fundamental building blocks and regulatory elements within cells. Metabolites are crucial in facilitating intercellular communication and orchestrating discrete physiological processes. The discernment and characterization of specific metabolites present a profound opportunity to enhance our understanding of disease progression, thereby enabling interventions at the foundational origins of pathological conditions (Qiu et al., 2023). The metabolome encompasses the collection of small-molecule metabolites within a cell during specific physiological conditions. The metabolome is a more time-sensitive indicator of perturbations, providing more accessible and accurate measurements than the transcriptome or proteome (Kell et al., 2005). Biofluids emerge as exceptional proxies for organs or tissues, given that their constituent elements closely mirror the metabolic activities of the originating organ or the organs they encapsulate (Evans et al., 2020). Ferroptosis is a form of programmed cell death first described in 2012 (Dixon et al., 2012). Recent advancements in mass spectrometry have enabled high-throughput analysis of metabolites, shedding light on ferroptosis regulation. Research on the role and regulation of ferroptosis in eye diseases remains scarce, with most investigations concentrating on retinal disorders like age-related macular degeneration and retinitis pigmentosa (Wei et al., 2024). Cysteine (Cys), crucial for glutathione (GSH) synthesis, supports glutathione peroxidase 4 (GPX4) in preventing ferroptosis, while ubiquinone or Coenzyme Q10 (CoQ10) metabolism inhibits ferroptosis independently of GPX4. Additionally, several PUFA-containing lipid enzymes can induce ferroptosis (Nguyen et al., 2022). Cells undergoing ferroptosis may exhibit elevated ECM production or release (Yan et al., 2024).

We methodically examined the intraocular fluid composition among individuals afflicted by PEXS during this investigation. Our emphasis lies in discerning potential disparities in the metabolic profiles of individuals manifesting PEXS. Although prior studies have explored pseudoexfoliation syndrome analysis in relation to aqueous humor, our objective is to conduct a larger population-based study aimed at identifying patterns that could provide insights into the underlying biochemical and pathological processes of this complex ocular pathology.

## 2 Materials and methods

### 2.1 Study group

The investigation encompassed the analysis of aqueous humor specimens derived from a cohort comprising 183 patients who had undergone cataract surgery at Pauls Stradins Clinical University Hospital. Within this selected cohort, 68 were male and 115 were female. The mean age of individuals diagnosed with cataracts and operated on was 73.6 years, with a standard deviation of ± 9.42. The age spectrum ranged from a minimum of 50 years to a maximum of 94 years. Notably, 104 patients lacked PEX, while 79 individuals manifested this ocular condition. It is crucial to emphasize that study participants who were presented with co-occurring ocular pathologies—such as diabetes, glaucoma, or age-related macular degeneration—were kept in the study population. Age-related macular degeneration data was not collected as part of this study, as it was not included in the study protocol. Therefore, the potential role of age-related macular degeneration in aqueous humor metabolomics was not assessed as a confounding factor as a limiting factor for our study. The demographic characteristics and comorbidities are shown in Table 1.

**TABLE 1:** Summary of demographics and ophthalmic co-morbidities among patients included in the study.

	PEXS (n = 79)	No PEXS (n = 104)	*p*-value
Age, mean (SD)	76.0 ± 8.24	71.8 ± 9.87	0.002
Gender			0.001
Male	33 (18.0%)	35 (19.1%)	
Female	46 (25.1%)	69 (37.7%)	
Axial Length, median (SD)	23.4 ± 1.24	23.6 ± 2.67	0.032
Cataract hardness (SPONCS)			0.032
1 (Subcapsular with clear nucleus)	0 (0.0%)	3 (1.6%)	
2 (Mild hardness)	17 (9.3%)	40 (21.9%)	
3 (Moderate hardness)	40 (21.9%)	22 (12.0%)	
4 (Advanced hardness)	5 (2.7%)	17 (9.3%)	
5 (Hypermature/Morganian)	17 (9.3%)	22 (12.0%)	
Glaucoma			0.001
Present	27 (14.8%)	13 (7.1%)	
Absent	52 (28.4%)	91 (49.7%)	
Diabetes			0.146
Present	19 (10.4%)	27 (14.8%)	
Absent	60 (32.8%)	77 (42.1%)	

The t-test was used to compare distributions of age, the Mann-Whitney U test for nonparametric axial length, while the chi-squared test was employed to analyze associations among categorical variables.

A paracentesis was conducted before the surgical procedure started, and a 27G needle was used to biopsy the aqueous humor. The aqueous humor is a more suitable fluid for analysis when studying the changes occurring in the anterior chamber, as compared to blood. A volume ranging from 50 to 120 µL of aqueous humor was aspirated and then transferred to Eppendorf tubes, depending on the volume of the accessible fluid. Samples were stored on-site at −18°C and delivered the same day in an ice box to Riga Technical University’s Faculty of Materials Science and Applied Chemistry for further analytical work and archive storage at −80°C.

### 2.2 LC-MS based metabolite analysis

Metabolites were extracted using a methanol-based extraction protocol. 10 μL of the aqueous humor sample were transferred to an empty Eppendorf tube and mixed with 80 µL methanol and 10 µL isotopically labeled internal standards. Each sample was vortexed for 15 s and then centrifuged for 10 min at 10.000 RPM. The supernatant was transferred into an HPLC glass vial.

Targeted quantitative metabolite analysis was conducted using HILIC-based liquid chromatography combined with mass spectrometric detection employing a Thermo Orbitrap Exploris 120 mass spectrometer. An ACQUITY UPLC BEH Amide 1.7 μm 2.1 × 100 mm analytical column (Waters) was employed for chromatographic separation. The gradient elution was carried out using 0.15% formic acid and 10 mM ammonium formate in water as mobile phase A and a solution of 0.15% formic acid and 10 mM ammonium formate in 85% acetonitrile as mobile phase B. The initial conditions were set to 100% in mobile phase B. After 6 min, a 0.1 min gradient (6.0–6.1 min) was started, and the mobile phase B level was reduced to 94.1%. From 6.1 to 10 min, mobile phase B was set to 82.4%, and from 10 to 12 min, mobile phase B was set to 70.6%. The column was then equilibrated for 6 min at initial conditions. The total analysis time was 18 min. The mobile phase flow rate was 0.4 mL/min, the injection volume was 2 μL, and the column temperature was 40°C. We utilized a well-established method developed in our lab, building on the foundational work of Prinsen et al. (2016). This method has been widely applied in metabolomics and has been previously reported in multiple studies (Elbere et al., 2024; Fritsch et al., 2023).

For MS detection, an Orbitrap Exploris 120 (Thermos Fisher Scientific) mass spectrometer was used. The MS analysis was performed in ESI positive and ESI negative modes using full scan detection; the scan range was set from 50 to 600 m/z, and the mass resolution was set to 60,000. The ESI spray voltage was set to 3.5 kV in positive mode and 2.5 kV in negative mode; the gas heater temperature was set to 400°C; the capillary temperature was set to 350°C; the auxiliary gas flow rate was set to 12 arbitrary units; and the nebulizing gas flow rate was set to 50 arbitrary units. For quantitative analysis, seven-point calibration curves with internal standardization were used. Tracefinder 51.1 General Quan (Thermo Fisher Scientific) software was used for LC-MS data processing and quantification. Every reported metabolite was identified at level A (Alseekh et al., 2021) using an authentic standard compound previously mapped to the analytical system. Detailed information about metabolite identification (RT, m/z, HMDB IDs) is provided in the Supplementary Table S1. Many of the metabolites analyzed are commonly studied in metabolomic research and are associated with oxidative stress, energy metabolism, and amino acid metabolism (Liu X. et al., 2023).

### 2.3 Statistics

Metabolite concentrations from the targeted metabolomics analysis were analyzed with MetaboAnalyst 6.0 (Pang et al., 2024) and GraphPad Prism 9 (Mitteer and Greer, 2022). Before statistical analysis, data was log10 transformed, and every metabolite was scaled by mean-centering and divided by the square root of the standard deviation. This was done to make metabolites of different ranges statistically comparable using Gaussian generalized estimating equations (Bartel et al., 2013). The data before and after normalization and scaling is presented in the supplementary material. Significance tests were done using a nonparametric test. In addition, absolute measured concentrations of metabolites were plotted for bar plots to increase data transparency. P-values were calculated using the Mann-Whitney U test for nonparametric data, with the test selection based on the normality of the data distribution assessed by the Shapiro-Wilk test.

We performed multivariate analyses using MetaboAnalyst 6.0 to explore the overall data structure and identify potential outliers, employing Principal Component Analysis and Partial Least Squares-Discriminant Analysis. Outliers were identified through visualization of score plots, and their evaluation also considered potential biological variation. These findings suggest that outliers are likely to reflect biological variability rather than experimental errors. Consequently, the outliers were not removed from the study population to avoid compromising the validity of the data. The presence of outliers in metabolomic studies often reflects intrinsic variability within the population, underscoring the importance of considering interindividual variability when interpreting metabolomic data. These examinations are detailed in the supplementary material. Future studies could further explore these variations to uncover potentially meaningful subgroups within the study population.

## 3 Results

During our analysis of the study groups, we identified statistically significant differences between patients with PEX and those without, as detailed in Table 1. The average age of patients in the PEX group (76.0 ± 8.24) was significantly higher than in the non-PEX group (71.8 ± 9.87), with a mean difference of 4.23 years (95% CI [1.52 – 6.94]). This difference was statistically significant, t (181), *p* = 0.002, d = 0.460. This finding aligns with the well-established association between PEX and aging (Rumelaitiene et al., 2023). Aging is associated with changes in metabolic pathways related to oxidative stress and cellular senescence, which may partially overlap with those seen in PEX (Maldonado et al., 2023).

Gender association with PEXS (present/absent) was not statistically significant between males (41.8%/58.2%) and females (33.7%/66.3%), X^1^ (1) = 0.943, *p* = 0.331, Cramer’s V = 0.0832. While in our study females were more predominant than males, there is no clearly established gender predilection (Forsius et al., 2002). Notably, no follow-up research has yet explored PEX and its ophthalmological associations in the Baltic countries (Rumelaitiene et al., 2023).

Glaucoma association with PEXS (present/absent) was statistically significant in patients with glaucoma (34.2%/65.8%) and without glaucoma (12.5%/87.5%), X^1^ (1) = 11.1, p = < 0.001, Cramer’s V = 0.260. PEX is a well-established risk factor for the development of glaucoma (Jeng et al., 2007). Glaucoma has been linked to metabolic changes, including alterations in lipid and amino acid metabolism. While these factors may contribute to some of the observed changes, the discriminant metabolites identified align closely with the known pathophysiology of PEX, supporting their relevance to the disease (Rombaut et al., 2023). The potential confounding effects of age and glaucoma on metabolomic profiles cannot be entirely excluded. Future studies with larger cohorts and matched controls are needed to disentangle these influences.

**FIGURE 1:**
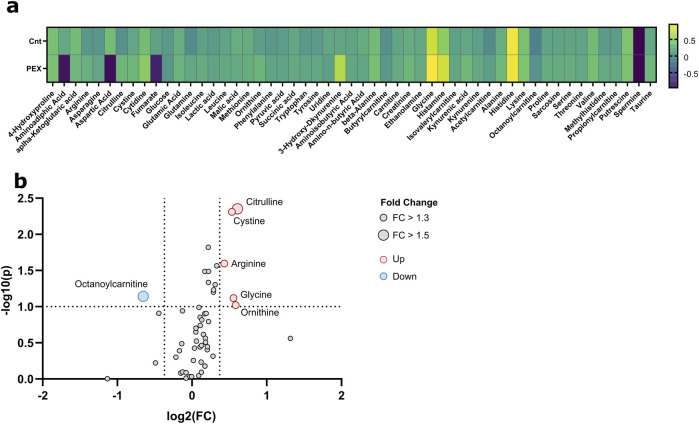
Metabolic analysis of aqueous humor samples from PEXS compared to control patients. **(A)** Heatmap of the relative changes of all quantified metabolites. **(B)** Volcano plots show fold changes (FC) and *p*-values between PEX and control patients; significant thresholds are FC > 1.3 and *p*-value <0.1 (dashed lines). Significant values are annotated.

**FIGURE 2:**
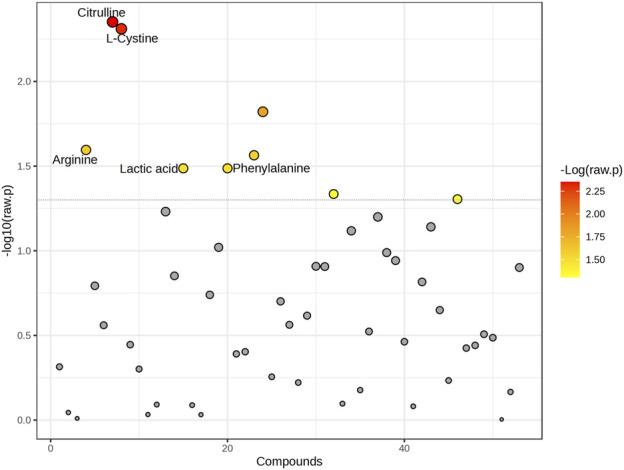
Graphical representation of all changed metabolites. The graph shows the -log10 (*p*-value) (*y*-axis) versus individual compounds (*x*-axis) analyzed in the study. Points are colored based on the -log10 (raw *p*-value), with red representing the most significant metabolites. The dotted horizontal line indicates the threshold for statistical significance. Key metabolites with notable differences, including *Citrulline*, *L-Cystine*, *Arginine*, *Lactic acid*, and *Phenylalanine*, are labeled for clarity.

Significant differences were observed in the metabolite concentration of aqueous humor between patients with and without PEXS. The intraocular cystine levels were notably higher in patients with PEXS (median = 124.5, IQR = 86.8–203) compared to those without PEXS (median = 96.35, IQR = 57.1–129), with a Mann-Whitney U test result of U = 2,317, *p* < 0.001, and an effect size of r = 0.306. Citrulline concentrations were similarly elevated in the PEXS group (median = 14.3, IQR = 8.08–23.0) versus the non-PEXS group (median = 8.26, IQR = 5.61–16.7), U = 2,492, *p* < 0.001, r = 0.303. Phenylalanine levels were also higher in the PEXS group (median = 100.9, IQR = 83.2–124) compared to the non-PEXS group (median = 91.2, IQR = 75.1–112), with U = 3,349, *p* = 0.041, r = 0.177. Similarly, tyrosine concentrations were greater in the PEXS group (median = 116, IQR = 88.5–143) than in those without PEXS (median = 102, IQR = 71.1–129), U = 3,245, *p* = 0.025, r = 0.195. Serine levels were elevated in patients with PEXS (median = 91.3, IQR = 67.5–117) relative to those without PEXS (median = 80.0, IQR = 51.2–109), U = 3,103, *p* = 0.030, r = 0.192. Additionally, arginine was significantly higher in the PEXS group (median = 556, IQR = 283–963) compared to the non-PEXS group (median = 343, IQR = 170–792), U = 3,108, *p* = 0.017, r = 0.209. Lactic acid concentrations were also elevated in PEXS patients (median = 5,202, IQR = 4,209–6937), relative to those without PEXS (median = 4,741, IQR = 3,695–6027) U = 3,395, *p* = 0.035, r = 0.181. Tryptophan showed a similar trend with higher levels in the PEXS group (median = 60.3, IQR = 40.3–89.7) compared to the non-PEXS group (median = 52.8, IQR = 30.6–75.8), U = 3,324, *p* = 0.055, r = 0.167. Lastly, creatinine concentrations were significantly higher in the PEXS group (median = 24.7, IQR = 18.2–32.4) compared to those without PEXS (median = 20.7, IQR = 14.7–26.8), U = 2,992, *p* = 0.022, r = 0.202. Graphical illustrations, including the metabolic analysis of aqueous humor samples from PEXS compared to control patients, are presented in Figures 1, 2.

**FIGURE 3:**
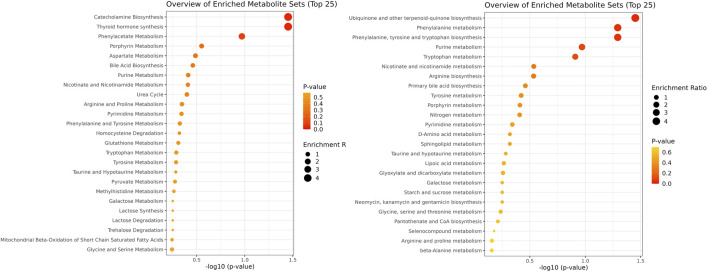
Pathway enrichment analysis using SMPDB and KEGG databases. The figure illustrates two pathways: SMPDB is represented on the left side, and KEGG is depicted on the right side.

**FIGURE 4:**
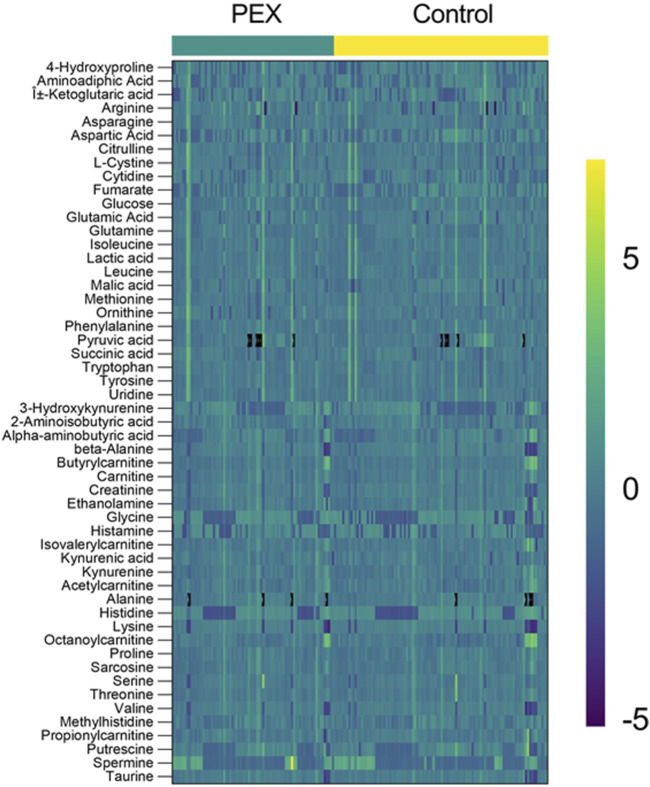
Heatmap of Metabolite profiles in PEX and control samples. The heatmap displays the relative abundance of metabolites in aqueous humor samples from PEXS patients (left, teal bar) and controls (right, yellow bar). Each row represents a metabolite (labeled on the left), and each column corresponds to an individual sample. The color scale ranges from purple (−5) indicating low relative abundance to green (0) representing median levels, and yellow (+5) showing high relative abundance. Distinct clustering patterns between the PEX and control groups highlight metabolite differences, emphasizing potential metabolic dysregulation in PEX.

Enrichment analysis showed a statistically significant difference in the ubiquinone and other terpenoid-quinone biosynthesis pathway based on the KEGG pathway, with a *p*-value of 0.0353, though only one metabolite was altered in this pathway. The phenylalanine metabolism and phenylalanine, tyrosine, and tryptophan biosynthesis pathways had low p-values of 0.0509 each, but these were not statistically significant. Results of enrichment analysis are shown in Figure 3. A heatmap depicting changes in metabolites between groups is presented in Figure 4.

## 4 Discussion

To elucidate our findings, we will conduct a thorough analysis of the identified metabolites, evaluating their significance and role in the PEXS progression. Our discussion will aim to clarify the relevance of these metabolites in the context of programmed cell death, emphasizing their potential implications in PEXS pathology.

In other metabolic studies, the variations in metabolomic profiles between aqueous humor and serum for most metabolites can be linked to the metabolic activity of the ocular tissues (Snytnikova et al., 2016). A notable distinction in the metabolomic compositions of human aqueous humor and cornea has been identified. The elevated levels of organic acids, purines, and GSH in the cornea can be attributed to their synthesis within the tissue (Snytnikova et al., 2017). Findings suggest that metabolomic analysis of aqueous humor and vitreous humor is more appropriate for estimating the post-mortem interval than serum analysis (Zelentsova et al., 2020). Metabolic pathway analysis in the Dmuchowska et al. study revealed that the identified metabolites engaged in eight distinct metabolic pathways, with cysteine and methionine metabolism and arginine and proline metabolism being the most prominently represented. Her research indicates that PEXS is associated with increased oxidative stress and inflammation, alongside disruptions in cellular respiration and mitochondrial energy production (Dmuchowska et al., 2021). According to Myer et al., neither arginine nor tryptophan proved significant in PEXS patients’ plasma. According to Myer et al., this implies that the metabolism of these amino acids in PEXS is affected locally rather than systemically (Myer et al., 2020). Leruez et al. reported increased octanoyl-carnitine levels in the plasma of patients with PEXS (Leruez et al., 2018). Multiple cholesterol esters, phosphatidylcholines, triglycerides, and ceramides were present in significantly higher concentrations in the aqueous humor of patients with PEXG compared to all other groups, according to Collao et al. (2022). Other researchers have posited that hyperhomocysteinemia is either a cause or consequence of PEX (Leruez et al., 2018). Our findings corroborate the hypothesis that cysteine synthesis predominantly occurs via the transsulfation pathway, likely attributable to inhibiting the antiporter system X_c_^−^. While studies on iron concentration in PEXS patients have yielded varying results, Talebnejad et al. (2021) reported that patients with PEXS exhibited reduced serum iron and zinc levels. In contrast, Cumurcu et al. (2006) found that serum levels of iron and copper were significantly elevated in the PEX group compared to the control group. Also, apohemoglobin has been identified in PEX material (Sharma et al., 2018). These discrepancies in iron concentration may contribute to the differing prevalence and characteristics of PEXS in various geographic populations (Aström and Lindén, 2007).

During our statistical analysis, we observed that age and the presence of glaucoma were also statistically significant factors among patients with PEXS. Although it is well-established that PEXS is closely linked to aging and glaucoma, it remains uncertain whether the changes identified in our results directly mirror those associated with these conditions or reflect distinct underlying mechanisms. Preliminary research has indicated that patients with glaucoma exhibit metabolic alterations in pathways related to ascorbic acid metabolism, fatty acid oxidation, and glutaminolysis (Wang Y. et al., 2021). Age and the presence of glaucoma are important factors that could influence the metabolomic profile observed in this study. Elderly populations often exhibit a wide range of metabolic variability due to physiological aging. While our study design did not allow for complete disentanglement of these factors, we acknowledge their potential impact and recommend that future research incorporate stratified or matched designs to minimize these confounding effects.

Cysteine is a building block of glutathione (GSH), which is composed of Cys, glutamate (Glu), and glycine (Anchordoquy et al., 2019). GSH is one of the most crucial small-molecule antioxidants in somatic cells (Lu, 2013). In plasma, Cys predominantly exists in its oxidized disulfide form, known as cystine (CySS). Furthermore, the increase in cellular GSH levels upon adding exogenous CySS suggests that CySS, once reduced to Cys within the cell, can be utilized for GSH synthesis (Noda et al., 2002). The antiporter system X_c_^−^ facilitates the import of CySS into cells while simultaneously exporting Glu in a 1:1 counter-transport ratio (Bannai, 1986). In 1980, Bannai and Kitamura first identified and characterized the antiporter system X_c_^−^ in cultured human fetal lung fibroblasts (Bannai and Kitamura, 1980). The system is a heterodimer consisting of the light-chain subunit SLC7A11 and the heavy chain subunit SLC3A2 (Huang et al., 2005). Mutations in different subunits SLC7A9 and SLC3A1 have been linked to cystinuria, an autosomal recessive disease characterized by the development of kidney stones (Pras et al., 1995). The antiporter system X_c_^−^ is critically involved in regulating several forms of programmed cell death, including ferroptosis, apoptosis, and autophagy-dependent cell death (Tu et al., 2021). The loss of the antiporter system X_c_^−^ leads to an oxidative shift in the aqueous humor, exposing the tissues interfacing with it to an elevated oxidative environment (Martis et al., 2020).

Ferroptosis enhances cellular susceptibility to lipid peroxidation (LPO) and iron-induced damage. The activity of the CySS/Glu antiporter system X_c_^−^, the synthesis of GSH, and the function of GPX4 collectively mitigate this vulnerability. These mechanisms preserve the integrity of essential metabolic pathways, including mitochondrial respiration, fatty acid metabolism, the mevalonate pathway, and selenium mercaptan metabolism (Björkegren and Lusis, 2022). In addition to being induced by the dysfunction of the antiporter system X_c_^−^, ferroptosis is often accompanied by inflammatory reactions (Yang Y. et al., 2022). Disruptions in iron metabolism can cause damage to macromolecules, including proteins, nucleic acids, and lipids, either through direct or indirect mechanisms (Andrews and Schmidt, 2007). Erastin, an inhibitor of the antiporter system X_c_^−^, induces GSH depletion by restraining CySS uptake and promoting ferroptosis (Yagoda et al., 2007). It can also be caused by the pharmacological inhibition of GPX4, for example, using RSL3 (Yang and Stockwell, 2008). The trans-sulfuration pathway can generate endogenous Cys to synthesize GSH when CySS import is inhibited (Zhu et al., 2019). The inhibition of the antiporter system X_c_^−^ results in elevated cystine levels in the aqueous humor.

Our pathway analysis, using KEGG, identified alterations in the biosynthesis of ubiquinone and other terpenoid-quinones. Research indicates that a reduction in intracellular ubiquinone levels can promote ferroptosis (Ren et al., 2023). The inhibition of lipid peroxidation by ubiquinone and the NAD(P)H-dependent oxidoreductase FSP1 at the plasma membrane safeguards cells from undergoing ferroptosis (Doll et al., 2019). These findings support the role of ferroptosis as a contributing factor in PEX pathology.

Oxidative stress can elevate the expression of pro-inflammatory cytokines, such as interleukin-6 and TNF-α, by activating crucial transcription factors like NF-κB (Hussain et al., 2016). When plasma membranes rupture, ferroptotic cells release intracellular components as danger signals for the innate immune system. These signals include products of LPO such as oxidized phospholipids, 4-hydroxynonenal (4-HNE), and prostaglandin E2, as well as damage-associated molecular patterns, like high-mobility group protein B1, DNA, and ATP (Zhang et al., 2023). The LPO product 4-HNE acts as a pro-inflammatory mediator by activating the NF-κB signaling pathway, contributing to the progression of chronic diseases (Jang et al., 2016). In individuals with PEXS and PEXG, there were increased concentrations of 4-HNE and 8-hydroxydeoxyguanosine, a widely used marker for DNA damage resulting from oxidative stress (Sova et al., 2010), observed in both the aqueous humor and serum (Koçak et al., 2023).

Lipoxygenases (LOXs) are enzymes that rely on either non-heme iron or manganese to catalyze the specific dioxygenation of 1Z,4Z-pentadiene units within polyunsaturated fatty acids (PUFAs), producing hydroperoxy fatty acids (Chrisnasari et al., 2022). LOXs and cytochrome P450 oxidoreductase (POR) have been identified as the major enzymes responsible for catalyzing LPO (He et al., 2022). Intracellular bioactive iron facilitates LPO by mediating the Fenton reaction and/or sustaining the enzymatic activities of LOXs and POR (Wang L. et al., 2021).

Arachidonic acid and adrenic acid containing phosphatidylethanolamines are particularly susceptible to ROS attack and thus serve as primary substrates for LPO. These long-chain PUFAs are preferentially converted into their acyl-CoA esters by acyl-CoA synthetase long-chain family member 4 (Doll et al., 2017). Supplementing cells with PUFAs promotes ferroptosis, whereas monounsaturated fatty acids (MUFAs) suppress ferroptosis by inhibiting LPO (Tesfay et al., 2019). The thiobarbituric acid reactive substances (TBARS) assay has been extensively utilized to measure LPO in biological fluids (Aguilar Diaz De Leon and Borges, 2020). TBARS, which are major breakdown products of lipid peroxides, are significantly elevated (by 200%) in the aqueous humor of patients with PEXS (Gartaganis et al., 2005).

Lactate dehydrogenase (LDH) is an enzyme that plays a crucial role in glycolysis. It facilitates the conversion of pyruvate (Pyr) to lactic acid when oxygen levels are low, and *vice versa* under aerobic conditions. Located in the cytoplasm, LDH activity increases outside the cell during oxidative stress due to cell membrane damage caused by LPO. This leads to higher levels of lactic acid (Jovanovic et al., 2010). Cys can be converted into Pyr, releasing free sulfate as a by-product. This conversion occurs through Cys catabolism, where Cys is first oxidized to cysteine sulfinate, which can be further metabolized into Pyr and inorganic sulfate. This process is significant in various tissues, including the eye, where the free sulfate might accumulate (Stipanuk and Ueki, 2011). Sulfation of proteins is a post-translational modification that influences protein-protein interactions, enzyme activity, and receptor binding. For instance, sulfation enhances the binding affinity of specific proteins to their ligands, which is vital in cellular communication and signal transduction pathways (Yang et al., 2015).

Tyrosine was also the first amino acid described when staining PEX material (Dvorak-Theobald, 1954). Within a peptide, tyrosine can undergo post-translational modifications such as nitration, phosphorylation, or sulfation, ultimately influencing the protein’s function. Tyrosine sulfation stands out as a distinct post-translational modification found in secreted and membrane-bound proteins of multicellular eukaryotes. In contrast to many other enzyme-catalyzed modifications, tyrosine sulfation is considered irreversible (Chen and Tsai, 2022). Tyrosine-sulfated proteins fulfill three roles: to integrate as standalone components of the extracellular matrix, to directly reattach to cells, or to indirectly reattach to cells via interactions with membrane-bound proteins (Kanan and Al-Ubaidi, 2015). Our hypothesis suggests that tyrosine-sulfated peptides are responsible for forming fibrillar material in PEXS syndrome. Immunoreactivity for keratan sulfate and dermatan sulfate proteoglycans has been observed within the PEX material deposited on the anterior surface of the lens capsule (Winkler et al., 2002).

Tryptophan (Trp) is needed for organisms’s responses to dietary and environmental signals (Cervenka et al., 2017). Tryptophan catabolism occurs through two main pathways. One, the serotonin pathway, involves tryptophan hydroxylase and produces serotonin (5-HT), a precursor for melatonin. The second pathway involves the conversion of Trp to kynurenine (Kyn) (Tsuji et al., 2023). 5-HT and 3-hydroxy anthranilic acid (3-HA) significantly enable tumor cells to evade ferroptosis through mechanisms distinct from Cys-mediated ferroptosis inhibition (Liu D. et al., 2023). Indoleamine 2,3-dioxygenase (IDO) is essential for tryptophan catabolism, explicitly starting the kynurenine degradation pathway (Mbongue et al., 2015). IDO induces ferroptosis by inhibiting the antiporter system X_c_^−^ (Yang J. et al., 2022). Notably, the activation of IDO is commonly evaluated using the Trp/Kyn ratio, which was not elevated in our study. Ultraviolet-B and ultraviolet-C irradiation catalyze tryptophan oxidation (Takikawa et al., 1986). Notably, increased time spent outdoors during youth is identified as a risk factor for PEXG (Leruez et al., 2018). Trp conversion is markedly augmented through the activation of IDO by interferon-γ, nitric oxide (NO), other cytokines, or superoxide anions (Nagalaxmi et al., 2016). Higher levels of IDO due to ultraviolet light and NO inhibit the antiporter system X_c_^−^.

Arginine serves as a metabolic precursor for several bioactive metabolites, including NO, urea, creatine, polyamines, proline, glutamate, guanabutamine, and hyperarginine, each of which is involved in various physiological processes (Martí and Reith, 2021). Arginine activates the mammalian target of rapamycin complex 1 (mTORC1) upstream of the Rag family of GTPases, either through the lysosomal amino acid transporter SLC38A9 or via the GATOR2-interacting CASTOR1 (Guo et al., 2023). Disruption of mTORC1 regulation is closely linked to various diseases, such as diabetes, cancer, and neurodegenerative disorders (Takahara et al., 2020). Arginine is critical for ferroptosis induced by erastin but not for ferroptosis induced by RSL3, as observed in MEF and HT1080 cells. Elevated arginine concentrations contribute to diminished intracellular GSH levels by promoting fumarate synthesis. Fumarate functions as a reactive, α,β-unsaturated electrophilic metabolite, covalently binding to GSH, forming succinic GSH. This interaction subsequently impairs antioxidant activity (Guo et al., 2023).

Citrulline (Cit) is synthesized by ornithine carbamoyl transferase and functions within the urea cycle via arginine succinyl synthetase (ASS). In NO producing cells, citrulline facilitates the synthesis of arginine through ASS, thereby contributing to the Cit-NO cycle. NO is a crucial inflammatory mediator, and its excessive production can exacerbate cardiovascular stress (Curis et al., 2005). Cit exerts regulatory control over NO synthesis through a negative feedback mechanism, thereby mitigating organ damage associated with oxidative stress (Gough et al., 2021). The intraocular fluid concentrations of NO are elevated in eyes with PEXS and PEXG compared to those in control subjects (Borazan et al., 2010).

Cit treatment has been demonstrated to significantly inhibit the expression of inflammatory cytokines, including TNF-α, IL-6, and IL-β. Moreover, the p65-dependent NF-κB signaling pathway is notably suppressed. These results indicate that Cit can attenuate inflammation in the thymus by mitigating the NF-κB signaling pathway activation induced by iron overload (Yin et al., 2023). PEX material contains traces of ferritin, although at a significantly lower concentration than the gelatin coating (Davanger, 1978).

Our findings suggest that ferroptosis may be implicated in the pathogenesis of PEXS, though further validation in in vitro models is necessary to confirm this hypothesis. The findings of this study should be interpreted with caution, as potential confounding factors, such as age and the presence of glaucoma, may have influenced the observed metabolomic patterns. Addressing these factors in future studies will be essential to further validate and refine these results.

Myopia is a common refractive error that has been increasingly linked to changes in the ocular metabolome, including the aqueous humor profile. Several studies have demonstrated that myopia, particularly high myopia, can influence the biochemical composition of aqueous humor, potentially contributing to changes in intraocular pressure and the development of associated ocular comorbidities (Grochowski et al., 2020). While our study does not directly address the relationship between myopia and aqueous humor metabolomics, it is important to acknowledge that myopia could represent a confounding factor in studies of aqueous humor biomarkers.

We find that PEXS may reflect ferroptosis in the anterior eye segment and in any cell expressing the antiporter system Xc^−^. This indicates that therapeutic strategies could extend beyond ocular treatment to encompass broader aspects of the patient’s health. A potential approach to managing pseudoexfoliation could involve lowering oxidative stress within the anterior chamber by reducing the uptake of PUFAs, lowering iron levels, and supplementing cysteine with stable forms like N-acetyl cysteine. Additionally, reducing dietary intake of PUFAs may help decrease lipid peroxidation and support the patient’s overall health. These strategies could contribute to improved ocular health and general wellbeing.

Despite the small size of our study population, our findings align with those reported by other researchers. We suggest that future studies replicate our methods to further explore these results and validate our conclusions. While this analysis is not intended to provide definitive guidelines for patients with PEXS, it represents a meaningful step forward in understanding the complexities of this ocular pathology.

## Data Availability

The original contributions presented in the study are included in the article/Supplementary Material, further inquiries can be directed to the corresponding author.

## Abbreviations

PEXS, pseudoexfoliation syndrome; ECM, extracellular matrix; PEX, pseudoexfoliation; ROS, reactive oxygen species; PEXG, pseudoexfoliative glaucoma; LOXL1, lysyl oxidase-like 1; CVD, cardiovascular disease; Cys, cysteine; CySS, cystine; Glu, glutamate; GPX4, glutathione peroxidase 4; RSL3, RAS-selective lethal 3; LPO, lipid peroxidation; 4-HNE, 4-hydroxynonenal; LOXs, lipoxygenases; PUFAs, polyunsaturated fatty acids; POR, cytochrome P450 oxidoreductase; MUFAs, monounsaturated fatty acids; TBARS, thiobarbituric acid reactive substances; LDL, low-density lipoprotein; Pyr, pyruvate; LDH, lactate dehydrogenase; mTORC1, mammalian target of rapamycin complex 1; Trp, tryptophan; Kyn, kynurenine; 5-HT, 5-hydroxytryptamine or serotonin; IDO, Indoleamine 2,3-dioxygenase; NO, nitric oxide; Cit, citrulline; ASS, arginine succinyl synthetase.
