# Supplementary material for: Aqueous humor metabolomic profiling identifies a distinct signature in pseudoexfoliation syndrome
Source: Front Mol Biosci. 2025 Jan 23;11:1487115. doi: 10.3389/fmolb.2024.1487115 (PMC11798801; doi:10.3389/fmolb.2024.1487115)
Supplement: Supplementary file 1 [file DataSheet2.pdf]

In this supplement, we present a comprehensive analysis of the data using several advanced statistical and computational techniques. First, we outline the results of data transformation and scaling, which were applied to standardize the dataset and ensure that all variables were on a comparable scale, reducing any biases due to differing units or ranges. Following this, we provide the outcomes of Principal Component Analysis (PCA), a technique used to identify patterns in the data by reducing its dimensionality and highlighting the most significant features that contribute to variability.

We also include the results from Partial Least Squares (PLS) regression, which was employed to model the relationship between the predictor variables and response variables, helping to reveal complex associations in the data. The variable importance in projection (PLS-VIP) scores are presented as well, which provide insights into the most influential features driving the PLS model.

Data transformation and scaling

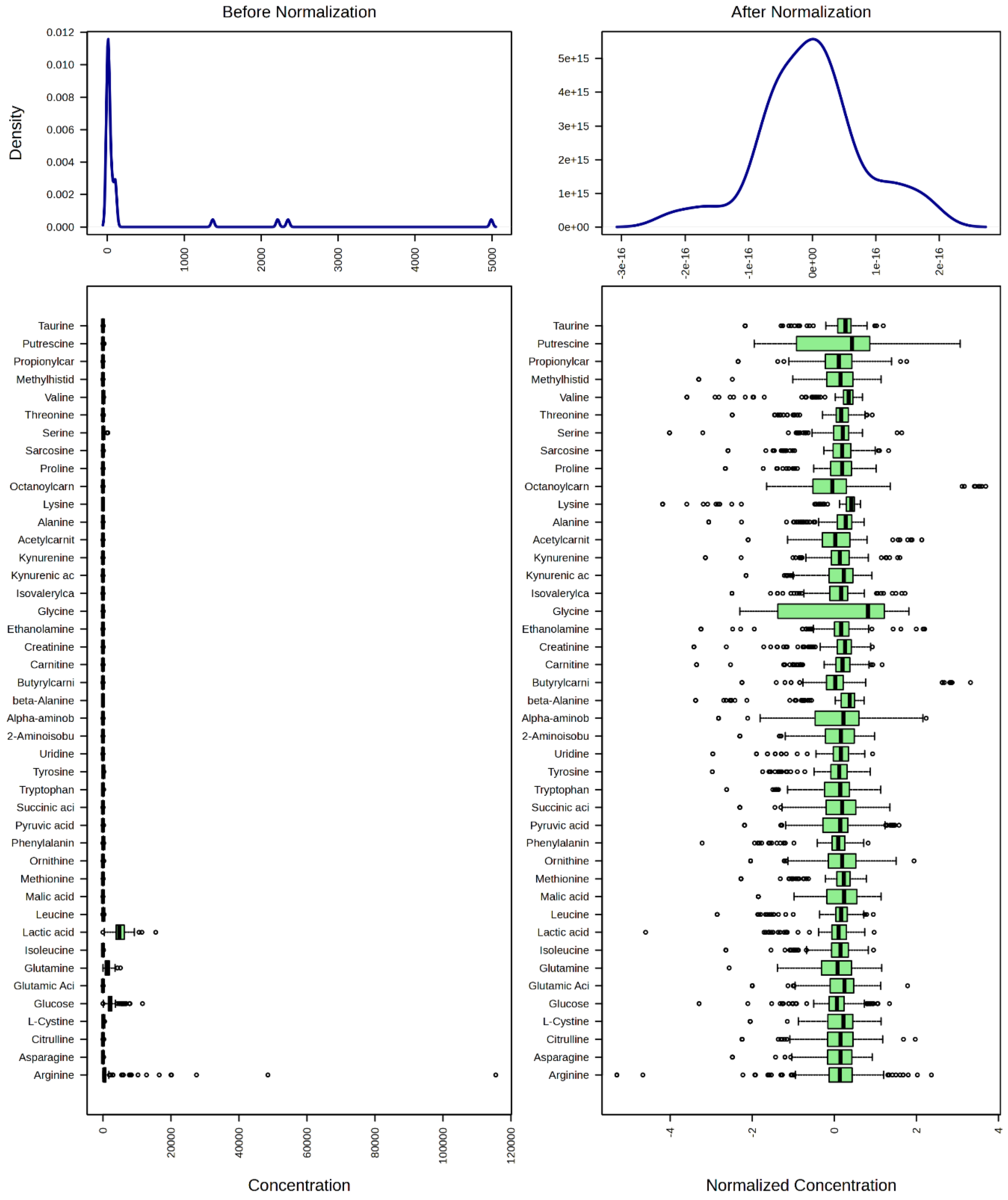

PCA

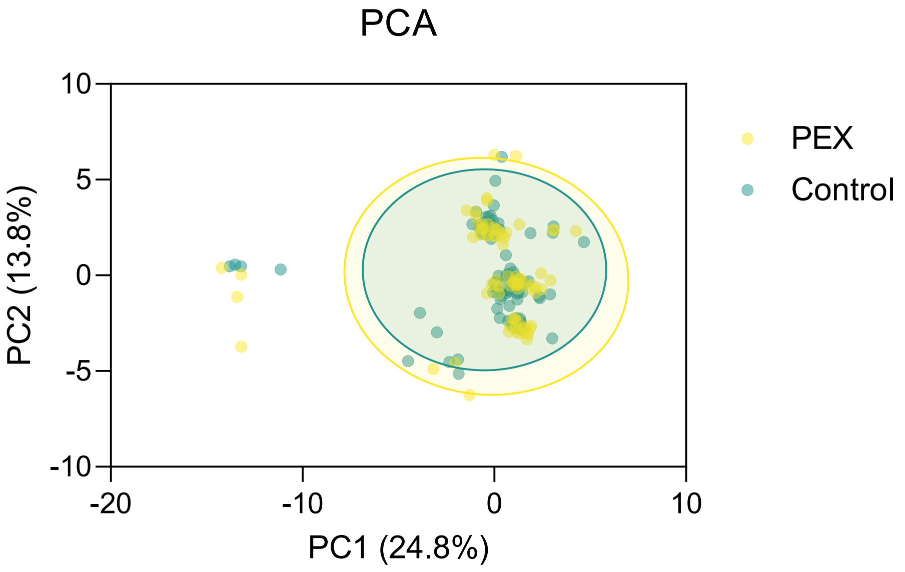

PCA
10
5
0
-5
-10
-20
-10
0
10
PC1 (24.8%)
PC2 (13.8%)
PEX
Control

PLS

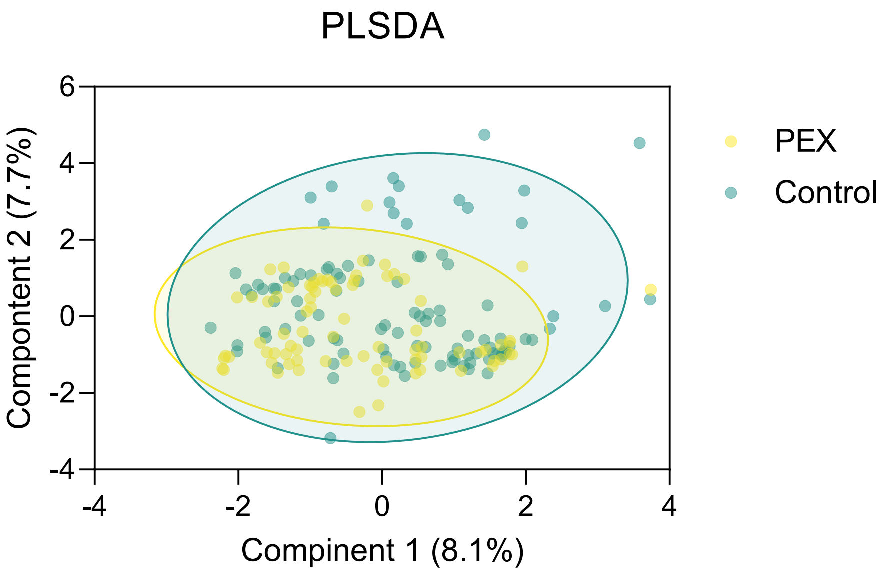

PLSDA
6
4
2
0
-2
-4
-4
-2
0
2
4
Compinent 1 (8.1%)
Compontent 2 (7.7%)
PEX
Control

PLS-VIP

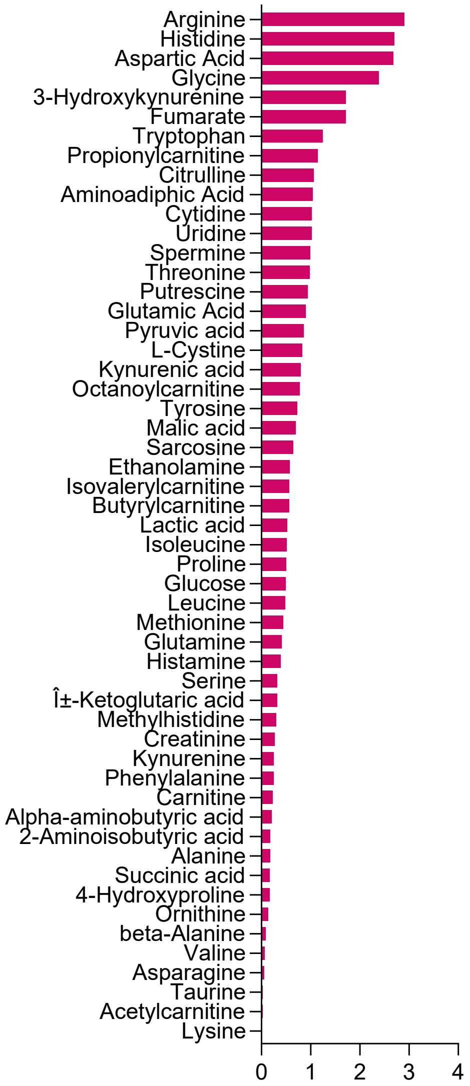
Arginine
Histidine
Aspartic Acid
Glycine
3-Hydroxykynurenine
Fumarate
Tryptophan
Propionylcarnitine
Citrulline
Aminoadiphic Acid
Cytidine
Uridine
Spermine
Threonine
Putrescine
Glutamic Acid
Pyruvic acid
L-Cystine
Kynurenic acid
Octanoylcarnitine
Tyrosine
Malic acid
Sarcosine
Ethanolamine
Isovalerylcarnitine
Butyrylcarnitine
Lactic acid
Isoleucine
Proline
Glucose
Leucine
Methionine
Glutamine
Histamine
Serine
Î±-Ketoglutaric acid
Methylhistidine
Creatinine
Kynurenine
Phenylalanine
Carnitine
Alpha-aminobutyric acid
2-Aminoisobutyric acid
Alanine
Succinic acid
4-Hydroxyproline
Ornithine
beta-Alanine
Valine
Asparagine
Taurine
Acetylcarnitine
Lysine
0
1
2
3
4
